# Multipolar Spindle Pole Coalescence Is a Major Source of Kinetochore Mis-Attachment and Chromosome Mis-Segregation in Cancer Cells

**DOI:** 10.1371/journal.pone.0006564

**Published:** 2009-08-10

**Authors:** William T. Silkworth, Isaac K. Nardi, Lindsey M. Scholl, Daniela Cimini

**Affiliations:** 1 Virginia Tech, Department of Biological Sciences, Blacksburg, Virginia, United States of America; 2 Department of Biology, Oberlin College, Oberlin, Ohio, United States of America; University of Edinburgh, United Kingdom

## Abstract

Many cancer cells display a CIN (*C*hromosome *In*stability) phenotype, by which they exhibit high rates of chromosome loss or gain at each cell cycle. Over the years, a number of different mechanisms, including mitotic spindle multipolarity, cytokinesis failure, and merotelic kinetochore orientation, have been proposed as causes of CIN. However, a comprehensive theory of how CIN is perpetuated is still lacking. We used CIN colorectal cancer cells as a model system to investigate the possible cellular mechanism(s) underlying CIN. We found that CIN cells frequently assembled multipolar spindles in early mitosis. However, multipolar anaphase cells were very rare, and live-cell experiments showed that almost all CIN cells divided in a bipolar fashion. Moreover, fixed-cell analysis showed high frequencies of merotelically attached lagging chromosomes in bipolar anaphase CIN cells, and higher frequencies of merotelic attachments in multipolar vs. bipolar prometaphases. Finally, we found that multipolar CIN prometaphases typically possessed γ-tubulin at all spindle poles, and that a significant fraction of bipolar metaphase/early anaphase CIN cells possessed more than one centrosome at a single spindle pole. Taken together, our data suggest a model by which merotelic kinetochore attachments can easily be established in multipolar prometaphases. Most of these multipolar prometaphase cells would then bi-polarize before anaphase onset, and the residual merotelic attachments would produce chromosome mis-segregation due to anaphase lagging chromosomes. We propose this spindle pole coalescence mechanism as a major contributor to chromosome instability in cancer cells.

## Introduction

Accurate mitotic chromosome segregation is necessary to maintain a diploid chromosome number. Most cancer cells are aneuploid [1], [2] and aneuploidy was suggested, already a century ago, to be a cause of cancer [3]. In addition to this aneuploid state, many cancer cells exhibit high rates of chromosome mis-segregation (i.e., gain or loss of whole chromosomes) at each cell cycle, a condition referred to as chromosome instability or CIN [4]–[6], which contributes to maintaining high levels of aneuploidy. A number of studies have tried to identify the defect(s) in mitotic chromosome segregation potentially responsible for CIN. Early studies suggested defects in the mitotic checkpoint as the main cause of CIN [7]. However, subsequent work has shown that most CIN cancer cells have a robust checkpoint [8] and their response to mitosis perturbing treatments is undistinguishable from the response of non-CIN cells [8], [9]. Cytokinesis failure has also been suggested in the past as a possible cause of CIN [10], [11]. However, cytokinesis failure would produce a single polyploid daughter cell, and could not explain CIN, which is defined as the mis-segregation of chromosomes at higher rates. As a result, CIN produces both high levels of aneuploidy and large variability in chromosome copy number within the population [4], whereas cytokinesis failure would simply result in a doubling of the chromosome number. Thus, cytokinesis failure *per se* cannot explain CIN, unless it is followed by other chromosome mis-segregation events in which single chromosomes (rather than the whole genome) are mis-segregated. Other studies suggested multipolarity as a potential cause of CIN [12]–[15] based on the observation that cancer cells from numerous sites (reviewed in [1]) frequently assemble multipolar spindles (usually accompanied by centrosome amplification). Although multipolar chromosome segregation would certainly lead to extensive chromosome mis-segregation, a number of studies suggested that many of these multipolar cells might undergo a process of spindle pole coalescence/clustering [16], [17], which would prevent the massive chromosome mis-segregation that would be associated with multipolar chromosome segregation. It has been suggested that this spindle pole coalescence mechanism would confer a selective advantage to cells whose aneuploidy levels would otherwise be so severe to result in cell death [18], [19]. Finally, a number of studies have found high frequencies of anaphase lagging chromosomes (i.e., chromosomes that do not segregate to the spindle pole, but lag behind at the spindle equator during anaphase) in various cancer cells, including oral cancer cells [20], [21], human breast cancer cells [22], [23], ovarian carcinoma cells [24], and colorectal cancer cells [22]. One of these studies [22] also showed that the lagging chromosomes were merotelically oriented (i.e., their kinetochore was bound to microtubules from both spindle poles rather than just one). In summary, many alternative mechanisms of CIN have been proposed over the years; however, a comprehensive theory of how CIN is perpetuated is still lacking, and it is not clear if any correlation between some of these mechanisms exists.

In this study, we used CIN colorectal cancer cells as a model system to investigate the possible cellular mechanism(s) underlying CIN. We found that CIN cells frequently assembled multipolar spindles in early mitosis, but multipolar anaphases were very rare, and almost all CIN cells divided in a bipolar fashion. We also found that bipolar anaphase CIN cells exhibited high frequencies of merotelically attached lagging chromosomes. Moreover, a significant fraction of bipolar metaphase/early anaphase CIN cells possessed more than one centrosome at a single spindle pole. Finally, we found high frequencies of merotelic attachments in multipolar prometaphases. Taken together, our data suggest a model by which merotelic kinetochore attachments can easily be established in multipolar prometaphases. Most of these cells would then bi-polarize before anaphase onset, and the residual merotelic attachments would produce chromosome mis-segregation due to anaphase lagging chromosomes. We propose this spindle pole coalescence mechanism as a major contributor to chromosome instability in cancer cells.

## Results

### CIN colorectal cancer cells possess multipolar spindles in prometaphase, but not in anaphase

**Figure 1:**
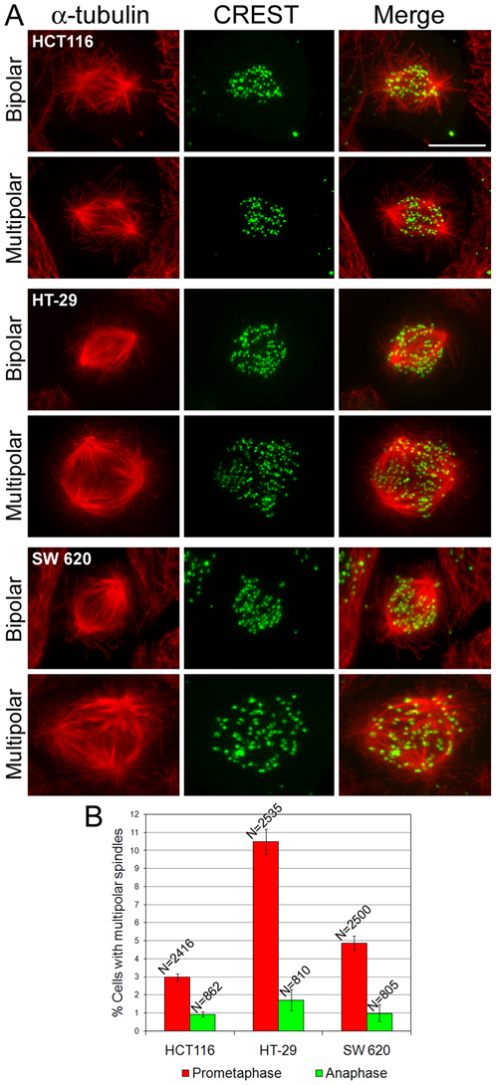
CIN cells frequently assemble multipolar spindles in early mitosis. A. Examples of MIN (HCT116) and CIN (HT-29, SW620) prometaphase cells immunostained for α-tubulin (red, left column) and kinetochores (green, middle column). All the images are maximum intensity projections of stacks of optical sections acquired at 0.6 µm intervals through the cell Z-axis. Merged images are shown in the right column. For each cell line, an example of bipolar prometaphase and one of multipolar prometaphase are shown. Scale bar, 5 µm. B. Frequencies of prometaphase and anaphase cells with multipolar spindles. The data shown here represent means and standard errors (bars) of four independent experiments. Multipolar CIN prometaphases were significantly more frequent than multipolar MIN prometaphases (χ^2^ test, P<0.001 for both HT-29 and SW620 when compared to HCT116). However, multipolar CIN anaphases occurred at low frequencies that did not differ from those of multipolar MIN anaphases.

Colorectal cancer cells can be divided in two groups [5], [25], those that exhibit CIN, and those that do not, traditionally named MIN because of their typical *M*icrosatellite *In*stability. Due to this characteristic, colorectal cancer cells represent a particularly interesting model for studying chromosome mis-segregation in cancer cells, because MIN cells can be used as an experimental control for CIN cells. For this study, we selected two CIN colorectal cancer cell lines (HT-29 and SW620) and one MIN colorectal cell line (HCT116). To identify mitotic defects potentially responsible for chromosome mis-segregation in CIN cells, we performed immunostaining experiments to label kinetochores (using CREST antibodies) and mitotic spindles (using anti-α-tubulin antibodies) in CIN and MIN cells. We then used high-resolution confocal microscopy to identify prometaphase defects in both CIN and MIN cells (Figure 1). We found that the most prominent prometaphase defect in CIN cells was spindle multipolarity and that CIN prometaphase cells exhibited multipolar spindles (Figure 1A) at frequencies that were significantly higher than those found in MIN cells (Figure 1B). Most of the multipolar spindles exhibited a tripolar or tetrapolar morphology, although multipolar cells with 5–8 spindle poles (5.4%, 19.5%, and 7.9% of all multipolar HCT116, HT-29, and SW620, respectively) were also observed. We then looked at multipolarity in anaphase cells, and found that the frequencies of multipolar spindles in anaphase CIN cells were much lower than those found in prometaphase (Figure 1B). In addition, there was no difference between MIN and CIN cells in the frequency of multipolar anaphases (Figure 1B).

### Both CIN and MIN cells divide in a bipolar fashion, but mitosis lasts longer in CIN cells

**Figure 2:**
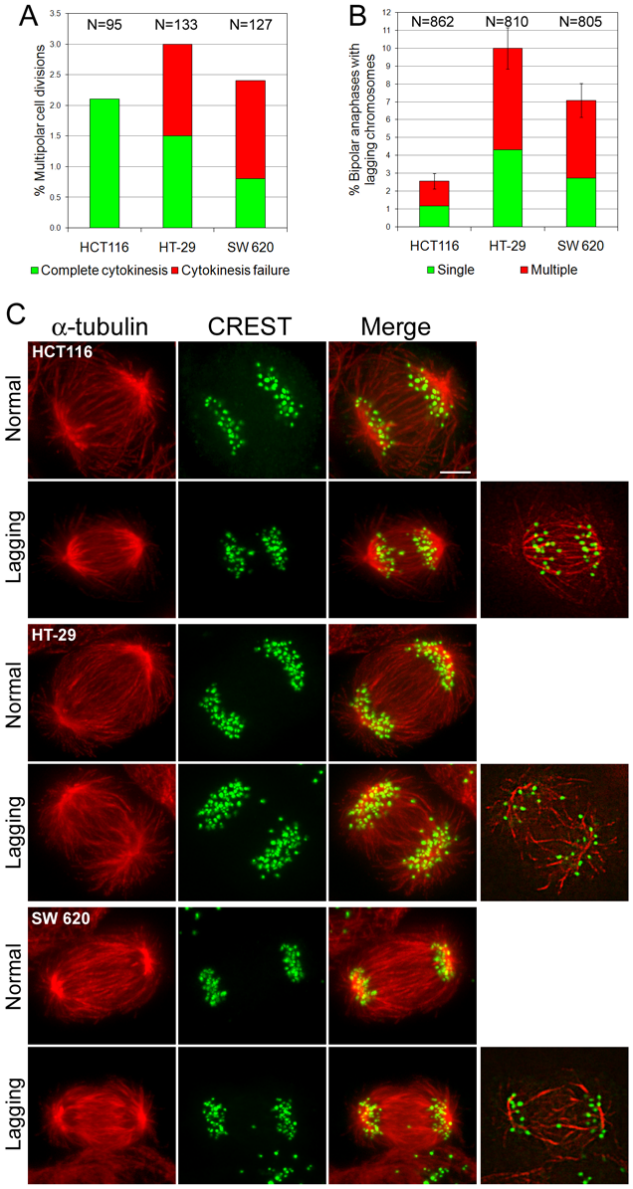
Most CIN cells divide in a bipolar fashion, but exhibit lagging chromosomes in anaphase. A. Frequencies of cells exhibiting multipolar chromosome segregation in phase-contrast time-lapse experiments. B. Frequencies of bipolar anaphase MIN (HCT116) and CIN (HT-29, SW620) cells with lagging chromosomes. The data shown here represent means and standard errors (bars) of four independent experiments. Frequencies of anaphase lagging chromosomes were significantly higher in CIN than MIN cells (χ^2^ test, P<0.001 for both HT-29 and SW620 when compared to HCT116). C. Examples of MIN (HCT116) and CIN (HT-29, SW620) anaphase cells immunostained for α-tubulin (red, first column) and kinetochores (green, second column). The images are maximum intensity projections of stacks of optical sections acquired at 0.6 µm intervals through the cell Z-axis. Merged images are shown in the third column. For each cell line, an example of normal anaphase and an anaphase with a lagging chromosome are shown. The column at the far right shows the cells with lagging chromosomes at a single focal plane, in which the contrast has been enhanced to highlight the merotelic connections of the lagging chromosome kinetochore. Scale bar, 5 µm.

The low frequencies of multipolar spindles in anaphase CIN cells suggested that multipolar prometaphases might not complete a multipolar division, but might instead encounter different fates. Some possibilities include mitotic arrest, mitotic cell death, or mitotic slippage. To determine the possible fate(s) of mitotic CIN cancer cells, we performed time-lapse experiments. In each experiment, multiple fields of view were selected and imaged by phase-contrast microscopy with a 20×objective on an inverted microscope equipped with a fully automated stage. Images were acquired every 30 sec for three hours. The time-lapse movies were subsequently analyzed to determine duration of mitosis and fate of cells entering mitosis during the period of recording. As expected from our fixed-cell data (Fig. 1B), we rarely observed chromosome segregation to occur in a multipolar fashion (Figure 2A). In most cells, chromosomes segregated into two groups at opposite sides of the cell, and one single cytokinetic furrow formed between them (Video S1). The number of cells exhibiting multipolar chromosome segregation in our live-cell experiments (Figure 2A) was not significantly different (χ^2^, P>0.37 for all three cell lines) from the number of multipolar anaphase cells we found in fixed cell experiments (Figure 1B). In addition, we found that 40–67% of the CIN cells exhibiting multipolar chromosome segregation failed to complete cytokinesis (Figure 2A), whereas there were no cases of cytokinesis failure in cells exhibiting bipolar chromosome segregation. Finally, we did not find any indication of persistent mitotic arrest, cell death during mitosis, or mitotic slippage. These data indicate that most of the chromosome instability in CIN cells must derive from chromosome segregation defects in cells dividing in a bipolar fashion and completing cell division. Our live-cell experiments also revealed that the time spent in mitosis, measured as the time elapsed from onset of cell rounding to anaphase onset, was significantly longer in CIN cells compared to MIN cells (Table 1), similarly to what others have found in other cancer cell lines [26].

**Table 1:** Duration of mitosis measured as time elapsed from onset of cell rounding to anaphase onset.

Cell line	Cell rounding – Anaphase onset (Mean±S.D.)	N
HCT116	23.29±6.34 min	135
HT-29	30.74±8.90 min*	135
SW 620	64.24±29.8 min*	135

* t-test, P<0.001, when compared to HCT116.

### Bipolar anaphase CIN cells exhibit high frequencies of merotelically attached lagging chromosomes

As described above, multipolarity was common in CIN prometaphase cells, but it was rarely observed in anaphase (Figure 1B), and most CIN cells segregated their chromosomes in a bipolar fashion (Figure 2A). This indicated that multipolar chromosome segregation is an unlikely cause of chromosome instability in CIN cells, and suggested that errors occurring during bipolar chromosome segregation were the most likely cause of CIN. To identify such potential defects, we used high-resolution confocal microscopy to analyze anaphase cells with immunostained kinetochores and microtubules (Figure 2C). We found that bipolar CIN anaphase cells possessed merotelically attached lagging chromosomes (i.e., chromosomes that lagged behind at the spindle equator instead of segregating to the spindle pole, and whose kinetochore was bound to microtubule bundles from both spindle poles rather than just one; Figure 2C, right column) at higher frequencies than MIN cells (Figure 2B). Interestingly, the frequencies of anaphase cells with lagging chromosomes were very similar to the frequencies of multipolar prometaphase cells (compare Figure 1B with Figure 2B).

### Both multipolar and bipolar CIN cells possess multiple centrosomes

**Figure 3:**
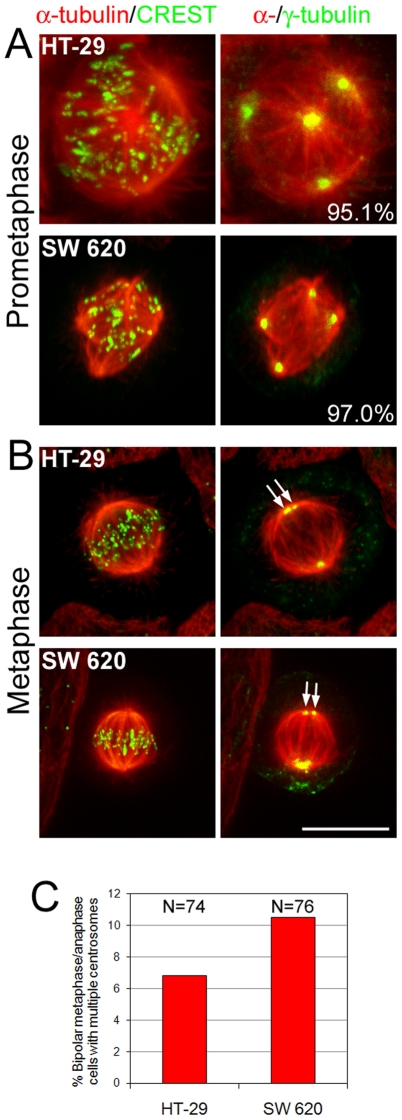
Both multipolar prometaphase and bipolar metaphase CIN cells possess multiple centrosomes. The figure shows examples of multipolar prometaphase (A) and bipolar metaphase (B) CIN cells immunostained for α-tubulin, kinetochores, and γ-tubulin. Images shown were obtained by merging maximum intensity projections of either α-tubulin and CREST (kinetochores) images (left column) or α-tubulin and γ-tubulin images (right column). A. Most multipolar prometaphase cells (exact percentages shown at the bottom right corner of the right panels) exhibited γ-tubulin staining at all spindle poles. B. Examples of bipolar metaphase CIN cells with multiple centrosomes at a single spindle pole (arrows point at γ-tubulin-stained dots). Scale bar, 5 µm. C. Frequencies of bipolar metaphase/early anaphase CIN cells possessing multiple centrosomes (as visualized by γ-tubulin staining) at a single spindle pole.

The low frequencies of multipolar anaphases compared to those of multipolar prometaphases (Figure 1B) and the bipolar chromosome segregation observed in live cells (Figure 2A) suggested that most of the multipolar spindles might bipolarize before anaphase onset. To test this hypothesis, we performed γ-tubulin staining in combination with microtubule and kinetochore immunostaining on CIN cells at different stages of mitosis (Figure 3A–B). High-resolution confocal microscopy revealed that in prometaphase CIN cells, γ-tubulin staining was present at all spindle poles in over 95% of the cells (Figure 3A). Next, we looked at bipolar metaphase/early anaphase cells (Figure 3B) and found that a significant number of cells exhibited multiple γ-tubulin-positive dots at a single spindle pole (Figure 3B–C), suggesting that some of the spindle poles present in multipolar prometaphase cells might move close together at later mitotic stages to generate two focused spindle poles, and hence a bipolar spindle. It should be noted that the observed frequencies (6.8% and 10.5% for HT-29 and SW620, respectively) could underestimate the actual number of spindle poles undergoing this coalescence process, as some of them might move so close to each other to appear as one by γ-tubulin staining.

### Higher frequencies of merotelic attachments in multipolar vs. bipolar prometaphase CIN cells

**Figure 4:**
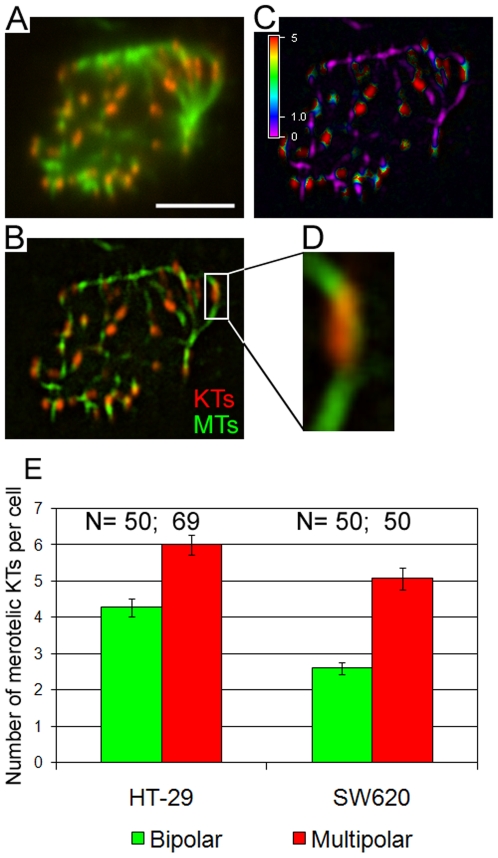
Multipolar prometaphase CIN cells possess larger numbers of merotelic kinetochores than bipolar prometaphase CIN cells. A. Single focal plane of an unprocessed HT-29 multipolar prometaphase. Scale bar, 2.5 µm. B. Same cell and same focal plane as in (A) obtained after processing of the images in the two channels by special filtering, merging, and smoothing (see Materials and Methods for details). A merotelic kinetochore is visible in the boxed area. KTs: kinetochores. MTs: microtubules. C. Ratio view of the focal plane shown in (A) and (B). Regions of juxtaposition between the kinetochore and its microtubule bundle(s) appear in green. D. Enlargement (400%) of the boxed area in (B). E. Average number of merotelic kinetochores in bipolar vs. multipolar prometaphase CIN cells. Multipolar prometaphases possess significantly more merotelic kinetochores than bipolar prometaphases (t-test, P<0.001 for both cell lines).

The multiple γ-tubulin signals at spindle poles of bipolar metaphase CIN cells, together with the occurrence of lagging chromosomes in bipolar anaphases at frequencies that closely resemble the frequencies of multipolar prometaphases, suggested that merotelic attachments might be preferentially formed in multipolar prometaphase cells that subsequently bi-polarize by spindle pole coalescence. To test this hypothesis, we used high-resolution confocal microscopy combined with 3-D visualization and image processing (see Materials and Methods for details) to identify merotelic kinetochores in cold-treated (to induce non-kinetochore microtubule disassembly, but preserve kinetochore microtubules) prometaphase CIN cells immunostained for kinetochores and microtubules (Figure 4A–D). We determined the number of merotelic kinetochores in bipolar vs. multipolar prometaphase CIN cells by identifying all the kinetochores bound to two microtubule bundles oriented in opposite directions, and found that multipolar prometaphase cells possessed significantly higher numbers of merotelic attachments than bipolar prometaphase cells (Figure 4E), suggesting merotelic attachments in such multipolar prometaphases as a major source of lagging chromosomes in bipolar anaphase cells.

## Discussion

### Cytokinesis failure does not contribute to CIN

Cytokinesis failure has been suggested in the past as a mechanism responsible for aneuploidy in cancer cells [10], [11]. Indeed, a number of studies have shown that polyploidy induced by experimentally inhibiting cytokinesis, can lead to malignant transformation and tumorigenesis [27], [28]. However, as pointed out in the introduction, cytokinesis failure *per se* would not be sufficient to explain CIN, i.e. high chromosome mis-segregation rates. Nevertheless, cytokinesis failure might be a contributing factor for CIN, thus we determined the frequency of cytokinesis failure both in cells exhibiting bipolar chromosome segregation and in cells with tripolar chromosome segregation. We never observed cytokinesis failure in cells undergoing bipolar cell division, and this phenomenon only occurred in about half of the cells undergoing multipolar cell division (Figure 2A). Because very few cells exhibit multipolar chromosome segregation, the observed rates of cytokinesis failure represent a very rare event in the overall cell population. Thus, cytokinesis failure fails to explain both the high rates of chromosome mis-segregation observed in CIN cells [4], [5] and the high rates of lagging chromosomes found in cancer cells [20]–[24] (Figure 2B). In conclusion, although cytokinesis failure could represent a rare, early event in tumor development [27], [28], it does not seem to contribute significantly to CIN in cancer cells at later stages of tumor progression.

### Multipolarity and multipolar chromosome segregation in CIN cancer cells

Many cancer cells have been previously shown to exhibit centrosome amplification [12], [15], [29], [30], and mitotic spindle multipolarity has been observed in cancer cells from various sites [1], [12], [15], [20], [21], [24], [29]–[34]. It has been suggested that multipolar chromosome segregation would produce largely aneuploid daughter cells, which would most likely not survive subsequent cell cycles [18], [19]. Indeed, our live-cell experiments showed that multipolar cell division is a rare event in CIN cells (Figure 2A). In addition, a recent study showed that multipolar cell division in CIN cells results in either cell death or cell cycle arrest [35]. It was previously suggested that multipolar cancer cells can undergo a bipolarization process, which would occur via centrosome clustering, and would lead to bipolar chromosome segregation [16]–[18]. Recent studies have shown that multiple players can be involved in promoting centrosome clustering (reviewed in [36]). These include actin-associated mechanisms [16], dynein [17], kinesin 14s (Ncd/HSET) [16], [37], and maybe other proteins/mechanisms [16], [36], [37]. Recent studies [16], [26], [37] have also suggested a possible role of the spindle assembly checkpoint in allowing time for spindle bipolarization in multipolar cells, as Mad2 inhibition prevented spindle bipolarization [16], [37] and accelerated mitotic exit in multipolar cells [26]. In agreement with these studies, we find that cells with higher frequencies of multipolar spindles spend, on average, longer times in mitosis (Table 1). However, Mad2 inhibition accelerates mitosis exit regardless of spindle pole number and kinetochore attachment [38]–[40], so its effect on multipolar spindle bi-polarization might simply be a secondary effect. We also found that multipolar cells possess higher numbers of merotelic kinetochores. However, merotelic attachments are not detected by the spindle assembly checkpoint (reviewed in [1]), and therefore this seems unlikely to be the reason for the mitotic delay. On the other hand, CIN cancer cells have excessive chromosome numbers, so it is easy to imagine that achieving stable attachments for all chromosomes will take longer in such cells compared to bipolar diploid cells. However, Yang et al. [26] recently showed that experimentally generated multipolar diploid RPE1 cells take twice as long to enter anaphase compared to their bipolar counterparts, suggesting that the increase in centrosome number might be enough to delay anaphase onset. The authors suggested that the presence of multiple microtubule asters might perturb the stability of kinetochore attachments [26], thus leading to an increase in the time necessary to complete kinetochore attachment, and consequently lengthening mitosis. This is an intriguing possibility, which will need to be tested in future experiments. In conclusion, how extra centrosomes delay anaphase onset is still unclear, but these studies suggest that both the extra chromosomes and the extra centrosomes in CIN cancer cells might contribute to the increase in average time spent in mitosis, during which the multipolar cells bipolarize via spindle pole coalescence. The centrosome clustering associated with spindle bi-polarization was previously suggested to confer cancer cells with a selective advantage that would prevent massive chromosome mis-segregation and allow cancer cells to keep dividing even in the presence of extra centrosomes [18], [19]. Thus, previous studies proposed such spindle pole coalescence process as a mechanism to prevent erroneous chromosome segregation. Conversely, we propose here that multipolar spindle assembly followed by spindle pole coalescence represents a major mechanism of chromosome mis-segregation in CIN cancer cells (see next section for a detailed description of our proposed model).

### Cancer cell multipolarity and merotelic kinetochore attachment: two sides of the same coin

The low number of multipolar anaphases compared to multipolar prometaphases (Figure 1B), the low number of multipolar cell divisions (Figure 2A), and the presence of multiple γ-tubulin-positive signals at single spindle poles in metaphase/early anaphase CIN cells (Figure 3B–C), suggest that most of the multipolar prometaphases undergo a process of spindle pole coalescence before anaphase onset, as previously suggested [16]–[19].

**Figure 5:**
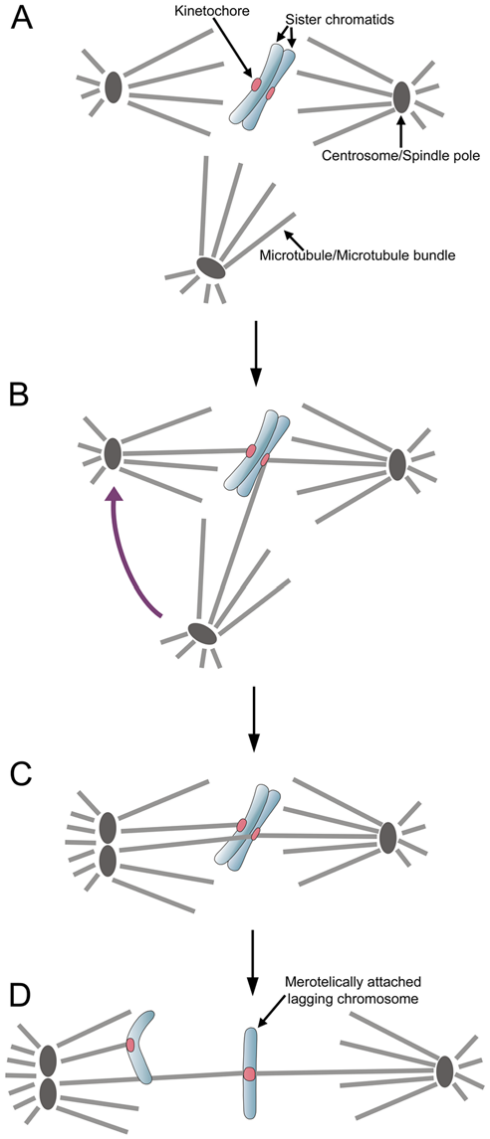
Schematic representation of the mechanism by which multipolarity can lead to merotelic kinetochore attachment and mitotic chromosome mis-segregation. A. Within a multipolar spindle, a single kinetochore is more likely to face two spindle poles than it would be in a bipolar spindle. B. Because of the multipolar spindle geometry, a single kinetochore can easily bind microtubules emanating from two spindle poles rather than from just one pole. After establishment of merotelic kinetochore attachment, the mitotic spindle bi-polarizes by a process of spindle pole coalescence (or centrosome clustering). C. Merotelic kinetochore attachment can persist through metaphase and into anaphase. D. During anaphase, the merotelic kinetochore attachment can give rise to a lagging chromosome.

We propose that when CIN cancer cells initially assemble multipolar spindles, single kinetochores are more likely than they would be within a bipolar spindle to face (Figure 5A), and become attached to (Figure 5B), two spindle poles (merotelic attachment). Thus, multipolar prometaphase cells would be expected to possess more merotelic attachments compared to bipolar prometaphases. Indeed, we found multipolar prometaphase cells to possess more merotelic kinetochores compared to bipolar prometaphases (Figure 4). As described above, multipolar spindles likely bipolarize via a spindle pole coalescence process (Figure 5B–C) before anaphase onset. Although correction mechanisms for merotelic kinetochore attachment exist [39], [41], [42], this kinetochore mis-attachment is not detected by the mitotic checkpoint [43], [44], and cells can enter anaphase before achieving complete correction [39], [43]. Because cells that transiently assemble multipolar spindles would start off with more merotelic attachments compared to bipolar cells (Figure 4 and 5B), more of such mis-attachments are expected to persist through anaphase and produce lagging chromosomes (i.e., chromosomes that lag behind at the spindle equator rather than segregating to the spindle pole; Figure 5D), and hence chromosome mis-segregation and aneuploidy. Remarkably, we also found that the frequencies of anaphase cells with merotelically oriented lagging chromosomes were very similar to the frequencies of multipolar prometaphase cells (compare Figure 1B and Figure 2B), suggesting the intriguing possibility that most lagging chromosomes are found in those cells that initially assemble multipolar spindles. In summary, whereas spindle multipolarity and anaphase lagging chromosomes had been previously suggested as unrelated causes of CIN, we show here for the first time that large numbers of merotelic kinetochores form in multipolar prometaphase cells, thus unveiling the close connection between multipolarity and anaphase lagging chromosomes.

We should note that, although at much lower frequencies than in CIN cells, we found both multipolar prometaphase spindles (Figure 1) and anaphase lagging chromosomes (Figure 2B–C) in HCT116 (MIN) cells. Nevertheless, these cells have been previously shown not to exhibit CIN [4], [22], and they were shown not to tolerate experimentally-induced chromosome mis-segregation [22], suggesting that CIN must result from a combination of high chromosome mis-segregation rates (not observed in HCT116 cells) and some other phenotypic feature that makes the cells tolerant for aneuploidy [22].

Future experiments should be aimed at testing our model and its prediction that inhibition of spindle pole coalescence should result in lower frequencies of lagging chromosomes in bipolar anaphase cells. Time-lapse imaging of cells with labeled kinetochores and microtubules would confirm the sequence of events proposed here (Figure 5) if merotelically attached anaphase lagging chromosomes were observed more frequently in cells starting mitosis with multipolar spindles compared to cells starting out with bipolar spindles. Moreover, spindle pole clustering could be inhibited to uncouple spindle multipolarity from anaphase lagging chromosomes, and thus demonstrate the causal relationship between these two phenomena. For instance, by performing a genome-wide RNAi screen in Drosophila S2 cells, Kwon et al. [16] found many different genes implicated in a variety of cellular processes, including spindle pole organization, cell shape and polarity, and cell adhesion, to be involved in centrosome clustering. According to our model, silencing of these genes in CIN cells should result in reduced frequencies of lagging chromosomes in bipolar anaphases, and future experiments should be aimed at testing this hypothesis. Although the reverse experiment (i.e., reducing merotelic attachment in multipolar cells) would be much more challenging, some experiments could provide indirect evidence that reducing merotelic attachment would result in a reduction in anaphase lagging chromosomes in cells that transiently assemble a multipolar spindle. For example, anaphase onset could be delayed by treatment of multipolar cells with a proteasome inhibitor. During the time of proteasome inhibition, the multipolar spindles are expected to bi-polarize, and the merotelic attachments are expected to be corrected. Thus, upon washout of the inhibitor, the cells should enter anaphase and exhibit low frequencies of lagging chromosomes.

### Does any other mechanism contribute to CIN?

We do not rule out that other mechanisms might contribute to chromosome instability in cancer cells. For instance, recent studies suggested that the mechanisms of merotelic kinetochore correction are not very efficient in CIN cancer cells [9], [45]. This means that CIN cells would exhibit slower kinetochore-microtubule turnover (required for correction of mis-attachments [42]) compared to MIN (or other chromosomally stable) cells. However, accurate comparison of microtubule dynamics in CIN vs. MIN (or other chromosomally stable) cells has not been performed. Even if such reduced correction efficiency were confirmed, however, it would simply add up to the increased number of kinetochore mis-attachments in cells that start mitosis with multipolar spindles (Figures 4 and 5). Moreover, there could be additional, as yet unidentified, mechanisms contributing to formation of large numbers of mis-attached kinetochores in CIN cancer cells. However, whether additional mechanisms exist or not, the mechanism described in this study could explain a large fraction of the chromosome mis-segregation occurring in CIN cancer cells.

## Materials and Methods

### Cell lines and culture conditions

All cell lines were obtained from the American Type Culture Collection. HCT116 and HT-29 cells were maintained in McCoy's 5A medium (Gibco), whereas SW620 cells were maintained in L-15 Medium (Gibco). All the media were supplemented with 10% fetal bovine serum, penicillin, streptomycin, and amphotericin B (antimycotic). All cell lines were grown in a 37°C, 5% CO_2_, humidified incubator. For experiments, cells were grown on sterile coverslips inside 35 mm Petri dishes.

### Immunostaining

Cells were rapidly rinsed in PBS, pre-fixed for 10 sec in 4% formaldehyde, permeabilized for 5 min in PHEM buffer containing 0.5% Triton X-100, and then fixed for 20 min in 4% formaldehyde. Subsequently, cells were washed in PBS, and then blocked in 10% boiled goat serum for 1 h at room temperature. The coverslips were then incubated overnight at 4°C in primary antibodies diluted in 5% boiled goat serum. Cells were finally washed in PBST (PBS with 0.05% Tween 20), incubated in secondary antibodies diluted in 5% boiled goat serum for 1 hour at room temperature, washed again, stained with DAPI, and mounted in an antifade solution containing 90% glycerol and 0.5% *N*-propyl gallate. For analysis of merotelic attachments in bipolar vs. multipolar cells, coverslips were first incubated in ice-cold medium (to disassemble non-kinetochore microtubules and preserve cold-stable kinetochore-microtubules) and kept at 4°C for 10 min; then they were processed as described above. Primary antibodies were diluted as follows: CREST (human anti-centromere protein, Antibodies Inc.), diluted 1∶100; mouse anti–α-tubulin (DM1A, Sigma-Aldrich), diluted 1∶500; rabbit-anti-γ-tubulin (Abcam), diluted 1∶100. Secondary antibodies were diluted as follows: X-Rhodamine goat-anti–human (Jackson ImmunoResearch Laboratories, Inc.), diluted 1∶100; Alexa 488 goat-anti–mouse (Molecular Probes), diluted 1∶400; Cy5-goat-anti-rabbit (Zymed Laboratories), diluted 1∶100.

### Confocal microscopy and image analysis

Immunofluorescently stained cells were imaged with a Swept Field Confocal system (Prairie Technologies) on a Nikon Eclipse TE2000-U inverted microscope. The microscope was equipped with a 100×1.4 NA Plan-Apochromatic phase–contrast objective lens, phase–contrast transillumination, transmitted light shutter, and automated ProScan stage (Prior Scientific). The confocal head was equipped with filters for illumination at 488, 568, and 647 nm from a 400 mW argon laser and a 150 mW krypton laser. Digital images were acquired with an HQ2 CCD camera (Photometrics). Image acquisition, shutter, Z-axis position, laser lines, and confocal system were all controlled by NIS Elements AR software (Nikon) on a PC computer. Z-series optical sections through each cell analyzed were obtained at 0.6 µm steps. Frequencies of multipolar prometaphases and anaphases, and anaphase lagging chromosomes were determined in 4 independent experiments, by viewing the samples via appropriate filter sets (Chroma Technologies). For experiments in which γ-tubulin staining was performed, each cell of interest was imaged as described above. For determination of the number of merotelic attachments in prometaphase cells, the acquired images were analyzed in multiple ways. First, both the kinetochore and microtubule images were processed through the “special filtering” function of the NIS Elements AR software to increase the contrast. These two processed images were then merged and smoothed (using the “smooth” function of the NIS Elements AR software). Merotelically attached kinetochores were then identified by scrolling through the Z-axis to visualize kinetochores bound to microtubule bundles oriented in opposite directions. When a merotelic kinetochore was identified, a “ratio view” was also created for that specific focal plane. This view allowed the identification of regions of juxtaposition between a kinetochore and its microtubule bundle(s). All the differently processed views of the image were simultaneously analyzed, and optical sections above and below were carefully examined to exclude all the cases in which a microtubule bundle ran past a kinetochore rather than ending on it.

#### Phase contrast live-cell imaging

Coverslips at ∼70% confluency were mounted into a Rose chamber [46] without top coverslip. The chamber was filled with L-15 medium with 4.5 g/l glucose, and mineral oil was added on top to prevent evaporation. Experiments were performed on a Nikon Eclipse TE2000-U inverted microscope equipped with phase–contrast transillumination, transmitted light shutter, ProScan automated stage (Prior Scientific), and HQ2 CCD camera (Photometrics). Cells were maintained at ∼36°C by means of an air stream stage incubator (Nevtek). Images were acquired and analyzed through the NIS Elements AR software. Images of ten different fields of view were acquired at 30 sec intervals over a three-hour period with an ADL 20×0.4 NA Achromatic phase-contrast objective. The time-lapse movies were subsequently analyzed to indentify cells undergoing mitosis during the period of recording. For each mitotic cell, complete progression through mitosis, chromosome segregation phenotype (bipolar or multipolar), and cytokinesis completion were determined by simply playing the time-lapse movie and observing the cell undergoing mitosis. In addition, mitotic timing was measured (like in [47]) as the time elapsed between onset of cell rounding and anaphase onset.

## Supporting Information

Video S1 HT-29 cells imaged every 30 seconds with a 20×0.4 NA ADL Achromatic phase-contrast objective. Three cells undergo mitosis during the time of recording. The three cells in this field of view all exhibit bipolar chromosome segregation.
(4.42 MB MOV)
